# The* in vitro* anti-fibrotic effect of Pirfenidone on human pterygium fibroblasts is associated with down-regulation of autocrine TGF-β and MMP-1

**DOI:** 10.7150/ijms.43238

**Published:** 2020-03-05

**Authors:** Yijin Tao, Qin Chen, Can Zhao, Xiao Yang, Qing Cun, Wenyan Yang, Yuan Zhang, Yingting Zhu, Hua Zhong

**Affiliations:** 1 Department of Ophthalmology, The First Affiliated Hospital of Kunming Medical University, Kunming 650032, China.; 2 Department of Ophthalmology, The First Affiliated Hospital of Nanjing Medical University, Nanjing 211166, China.; 3 Shandong Eye Hospital, State Key Laboratory Cultivation Base, Shandong Provincial Key Laboratory of Ophthalmology, Shandong Eye Institute, Shandong First Medical University & Shandong Academy of Medical Sciences.; 4 Tissue Tech, Inc., Miami, FL, 33126, USA.

**Keywords:** pirfenidone, human pterygium fibroblasts, proliferation, migration, collagen contraction, TGF-β, MMP

## Abstract

We aimed to investigate the *in vitro* effect of pirfenidone (PFD) on proliferation, migration and collagen contraction of human pterygium fibroblasts (HPFs). HPFs were obtained from tissue explants during pterygium surgery. After treatment with pirfenidone, the HPFs proliferation was measured by MTT, cell cycle progression measured by flow cytometry, cell migration measured by the scratch assay, and cell contractility evaluated in fibroblast-populated collagen gels. The expression of TGF-β1, TGF-β2, MMP-1 and TIMP-1 were also determined with quantitative PCR, western blot and immunofluorescence staining. Results showed pirfenidone markedly inhibited HPFs proliferation with an IC_50_ of approximately 0.2 mg/ml. After treatment with 0.2 mg/ml pirfenidone for 24 hours, HPFs were at G0/G1 cell cycle arrest, with significantly reduced cell migration capability and collagen contraction, decreased mRNA and protein expressions of TGF-β1, TGF-β2 and MMP-1, and no alterations of TIMP-1 expression. Thus, we have concluded that pirfenidone at 0.2 mg/ml inhibits proliferation, migration, and collagen contraction of HPFs, which is associated with decreased expression of TGF-β and MMP-1, and pirfenidone might represent a potentially therapeutic agent to prevent the recurrence of pterygium after surgery.

## Introduction

Pterygium is featured with non-cancerous growths of epithelial and fibrovascular tissue from the corneoscleral limbus. Pterygium is named because of its wing-shaped morphology, which is a common benign proliferation of epithelial and fibrovascular tissue from the corneoscleral limbus, and is characterized by an altered basal epithelial cell proliferation, vascularization, and invasion of the adjacent corneal epithelium 1,2. One of most significant risk factors for pterygium is the ultraviolet light exposure. As a result, people living in the equatorial and sun-exposed areas are preferably affected 3,4. The progression of pterygium causes irritation and affects visual function by disturbing the tear film, inducing astigmatism, or occluding the visual axis 1,2,5.

The most common treatment of pterygium is surgical resection. However, there is known to be a variable high rate of pterygium recurrence after surgery. Conjunctival autograft and amniotic membrane transplantation techniques have been employed in efforts to lower the recurrence rate. However they have their own limitations, including increased operation time, requirement of technical surgeon, and lack of amniotic membranes/fibrin glues availability 6-8. Intraoperative application of antifibrotic drugs such as mitomycin C (MMC) has also been used to reduce the recurrence rate 9,10. However, they are associated with high-risk complications, including delayed corneal epithelialization, prolonged postoperative corneal epithelial and stromal edema, and even corneal perforation 11-13. Therefore, new antiproliferative drugs with less toxicity and fewer complications need to be developed to effectively improve the success rate of the surgery. Such a development requires understanding the potential molecular mechanism of the occurrence and development of pterygium, which is of great importance for the study of non-surgical treatment strategies for pterygium and the prevention of postoperative disease recurrence 14.

Pirfenidone (5-methyl-1-phenyl-2-[1H]-pyridone, PFD) has its anti-fibrotic potential in animal models and clinical trials has been performed by down-regulating a series of cytokines, such as transforming growth factor beta (TGF-β) 15, connective tissue growth factor (CTGF) 16, platelet-derived growth factors (PDGF) 17 and tumor necrosis factor (TNF-α) 18. In addition, the anti-fibrotic activity and cellular safety of the agent have been demonstrated in tissues such as lung 19,20, liver 21 and kidney 16. Furthermore, PFD may inhibit proliferation, migration and collagen contraction in human tenon's fibroblasts by down regulating TGF-β signaling 20, and prevent scaring by inhibiting TGF-β and TIMP-1 pathways in experimental glaucoma surgery 21. Finally, pirfenidone also significantly inhibits fibronectin synthesis induced by TGF-β1 in human retinal pigment epithelial (RPE) cells 22.

Our team has been devoted to the investigation into the anti-scarring effects of pirfenidone and its possible clinical application after glaucoma filtration surgery. Previously, we reported that pirfenidone could prohibit the migration and proliferation of conjunctival fibroblasts both in vitro 23 and ex-vivo 24. In light of these findings and established roles of pirfenidone in fibroblast-matrix interplay 25,26, we hypothesized that pirfenidone could inhibit fibroblast proliferation and subsequent synthesis of extracellular matrix on primary and recurrent pterygium. Because we have discovered that PFD may inhibit proliferation, migration and collagen contraction in human tenon's fibroblasts by down regulating TGF-β signaling 20, and prevent scaring by inhibiting TGF-β and TIMP-1 pathways in experimental glaucoma surgery 21, we investigated the effects of PFD on proliferation, migration and collagen contraction of cultured human pterygium fibroblasts (HPFs), and further explored the potential molecular mechanisms. Our results indicate that pirfenidone at 0.2 mg/ml significantly inhibits proliferation, migration, and collagen contraction of HPFs. Further studies suggest that such inhibition is closely associated with decreased expression of TGF-β and MMP-1. Therefore, we conclude that pirfenidone might represent a potentially ideal therapeutic agent to prevent the recurrence of pterygium after surgery, which benefit the pterygium patients around the world.

## Materials and Methods

### Ethics statement

This study was approved by the Human Study Committee of the First Affiliated Hospital of Kunming Medical University (KMU), Kunming, China. All the experiments involving clinical samples were performed with adherence to the tenets of the Declaration of Helsinki. Written informed consent was obtained from all the participants in this study, approved by the ethics committees of KMU.

### Cell culture and identification

The pterygium tissues were obtained after routine resection, and all specimens used for pterygium fibroblasts cultures were taken from the head of pterygium. Human pterygium fibroblasts were established as previously reported 27. Briefly, the specimen was minced into small pieces and cultured for 7 days in DMEM medium (GIBCO, 11965) supplemented with 10% fetal bovine serum (FBS, GIBOCO, 16000036), 100 IU/ml penicillin, and 100 µg/ml streptomycin. The cells were maintained at 37°C with 5% CO2 in a humidified atmosphere. The medium was replaced every 3 days with fresh culture medium. When the cells reached 80% confluence, they were passaged at 1:2 or 1:3 ratios to new dishes after digestion with 0.25% trypsin. The cells of passages 6 to 8 were used for experiments. The stromal fibroblasts was confirmed by staining for vimentin and not for cytokeratin by confocal imaging as previously described 28 and the cell morphology were recorded by a phase-contrast microscope.

### Transfection of siRNAs

scRNA, siRNAs to MMP1, TGFβRI, TGFβRII and TGFβRIII (category numbers 4404021, 104015, 103324, 138859 and 107049) were obtained from ThermoFisher Scientific (Waltham, MA, USA). GeneEraser^TM^ siRNA transfection reagent was obtained from Stratagene (La Jolla, CA, USA).

Transfection was performed by adding 50 μl of serum-free, antibiotic-free DMEM to a polystyrene tube and 3 µl of GeneEraser siRNA transfection reagent followed by incubation at room temperature for 15 min. Three µl of 20 µM scrambled RNA (scRNA), siRNA to MMP1, siRNAs of TGFβRI, TGFβRII and TGFβRIII combinations was added to the above mixture, mixed gently by pipetting, and incubated for additional 15 min. The transfection mixture was added to a well of a 24-well dish with HPFs cultured in 250 µl of fresh medium. The dish was cultured at 37°C for 2 days in the incubator before proliferation (MMT assay).

### MTT assay

Pirfenidone and carboxymethylcellulose were purchased from Santa Cruz Biotechnology, USA. HPFs were divided into three groups as follows: Pirfenidone group (PFD, treated with PFD at IC_50_), blank control group (BC, without treatment) and negative control group (NC, treated with carboxymethylcellulose solution). MTT Cell Proliferation Kit was used to examine cell proliferation of HPFs (Abcam, Cambridge, MA, USA). All the procedures were performed following the instructions of the manufacturer. After cultured in 96-well plates for 24 hours in the incubator, HPFs were respectively treated with 0, 0.01, 0.1, 0.2, 0.3, 0.5 and 1 mg/ml pirfenidone (PFD) for 6, 12, 24, 48 and 72 hours. The absorbance of optical density (OD) at 570 nm in each well was read in a microplate reader (Bio-Rad, Munich, Germany). The inhibition ratio of cell growth (IR) was calculated via the following formula: (MTT OD value of control-MTT OD value of PFD treated cells)/MTT OD value of control] × 100%. The values of IC_50_ from HPFs were determined. Six replicates were performed for each concentration.

### Cell viability assay

Cell viability was determined by the trypan blue exclusion method. HPFs were treated with pirfenidone at the indicated concentration for 24 hours, collected and stained with 0.4% trypan blue solution. Stained (dead) and unstained (viable) cells were counted with a hemocytometer. The percentage of cell viability was calculated according to the formula: % of cell viability = (viable cell count/ total cell count) × 100%.

### Cell cycle assay by flow cytometry

HPFs were cultured on collagen-coated culture dishes with the complete DMEM medium supplemented with pirfenidone at the indicated concentration for 24 hours. The cells were trypsinized, collected, and fixed with 70% ethanol for 4 hours on ice. After fixation, the cells were treated with 50μg/ml RNase at 37°C for 30 minutes. Nuclei were stained by incubation with 100μg/ml propidium iodide (PI) at 4°C for 30 minutes. Data were acquired with a FACS Calibur flow cytometer (Becton-Dickinson, San Jose, CA, USA) and processed with the accompanying BD CellQuest™ Pro Software.

### Wound Healing Assay

Wound Healing Assay was used to measure the migration ability of HPFs (Abcam, Cambridge, MA, USA). When cells were grown to a confluent monolayer, serum was deprived for 24 hours. The medium was discarded, and a vertical scratch wound was inflicted across the cells with a p20 pipette tip. The suspended cells on the plates were washed by PBS twice. After that, the plates were incubated with the complete DMEM culture medium supplemented with indicated concentration of pirfenidone. Wound healing status was monitored and photographed under a light microscope and analyzed. The shortest distances between the edges of the cells migrating from both sides were measured.

### Migration assay

For migration assay, HPFs (1×10^5^/well) were grown on the filters of chambers with 8 µm pore size coated with Matrigel (Biocoat chambers, Becton Dickson, Bedford, MA, USA) for 24 h and then were treated with or without scRNA and MMP1 siRNA for 24 h, in the presence of transfection reagents. Cells on the upper surface of the filters were removed and cells adhering to the undersurface of the filters were counted. Each experiment was repeated 3 times.

### Fibroblast-mediated collagen contraction assay

Type I collagen was extracted and collagen gels were made as previously reported 29. After digested with Trypsin-EDTA and washed, HPFs were suspended in the complete DMEM medium at 5× 105 cells/ml. Aliquots (250μL) of a collagen/cell suspension mixture were pipette into single 24-well plates and allowed to polymerize. The gels were gently released from the walls of the well to allow contraction. Each lattice received treatment with 500μL/well of the indicated concentration for 7 days. The medium was changed every 3 days using the complete medium containing relative concentration of pirfenidone. Measurements of the fibroblast-populated collage lattice (FPCL) area were carried out with a digitizer (Olympus Technology, Tokyo, Japan), conjointly the calibration grids obtained from the photographs directly converted into an IBM-compatible computer. The areas from these digitized images were then calculated by the Sigmascan software (Jandel Scientific, Corte Madera, CA). The mean of triplicate lattices was used for statistical analysis.

### RNA Extraction and Reverse Transcription-quantitative Polymerase Chain Reaction (RT-qPCR)

Total RNA was isolated from cultured cells by acid guanidium thiocyanate-phenol-chloroform extraction. Reverse transcription was performed with the ThermoScript™ Reverse Transcriptase system (Fermentas, Burlington, ON, Canada). Approximately 1μg RNA and random primer were used in each reaction. The SYBR Green based real-time RT-PCR assay was performed using a SYBR PrimeScript RT-PCR Kit (TaKaRa, Dalian, China) following the instruction of the manufacturer. Diluted cDNA (0.5μg) was used for amplification in a 25μL PCR reaction volume. PCR was performed in a DNA thermal cycler (GeneAmp 2400; Perkin Elmer), with an initial denaturing step at 95°C for 10 minutes, followed by cycles of denaturation (95°C, 15 seconds), annealing (60°C and 40 cycles for 60 seconds), and extension (60°C, 60 seconds). Quantifications of signal intensity were confirmed using a specific computer program (IBAS2.5 Auto Image analysis; Kontron, Eching, Germany). The fidelity of RT-qPCR fragments was subsequently verified by examining the size of the amplified products with DNA gel electrophoresis and by DNA sequencing of the PCR products. Specific primers for detection of genes TGF-β1, TGF-β2, MMP-1 and TIMP and β-actin are listed in Table 1.

### Western blot

Cells were lysed with the 1×SDS lysis buffer. Protein concentrations of the cell lysates were determined using a BCA protein assay kit (Sigma-Aldrich, USA). 30μg proteins from each sample were resolved by 10% sodium dodecyl sulfate polyacrylamide gel electrophoresis (SDS-PAGE) and electrophoretically transferred to PVDF membranes. After blocking with 5% bovine serum albumin (BSA), the membranes were probed with anti-TGF-β1, anti-TGF-β2 (1:500 dilution, Cell Signaling, Beverly, MA, USA), anti-MMP-1, anti-TIMP-1 (1:1000 dilution, Cell Signaling, Beverly, MA) and anti-β-actin (1:1000 dilution, Cell Signaling, Beverly, MA) antibodies at 4°C overnight. After washing, the membranes were then incubated with horseradish peroxidase-conjugated secondary anti-rabbit sera (1:5000 dilutions, Dako, Ham-burg, Germany) in PBS with 0.5% Tween-20. Blots were developed by chemiluminescence, which produced signals that were captured on X-ray films (Eastman Kodak, Rochester, NY), according to the manufacturer's instructions. Membranes or chemiluminescent films were then scanned for densitometric analysis by the ImageJ software (National Institutes of Health, Bethesda, MD; available at http://rsb.info.nih.gov/ij/index.html).

### Immunofluorescence staining

Cells on slides were fixed in 4% paraformaldehyde/PBS for 30 minutes and rinsed with PBS. After permeabilization with 0.1% Triton X-100/PBS for 10 minutes, the slides were blocked in 3% BSA for 30 minutes. The slides were then incubated with the primary antibodies (anti-MMP1, Catalogue number ab52631, Abcam, 1:100 dilution; anti-TIMP1, ab1827, Abcam, 1:100 dilution; anti-TGFB1, BA0290, Boster, 1:50 dilution; anti-TGFB2, BA0292, Boster, 1:50 dilution; 100μl each) for 2 hours at room temperature. After washing with PBS three times, the slides were incubated with Fluorescein isocyanate (FITC)-conjugated anti-rabbit antibody at room temperature for 1 hour. After mounting (UltraCruz Mounting Medium, SC-24941; Santa Cruz Biotechnology, Inc., USA), the slides were subjected to image acquisitions under a laser scanning microscope (Leica TCS SP5, Leica Microsystems, Exton, PA). The images were then analyzed using the Image-Pro Plus 6.0 (IPP 6.0) software (Media Cybernetics, USA).

### Statistical analysis

Statistical analysis was performed using the SPSS software (version 19.0, IBM, USA). All results are expressed as the mean ± SD (standard deviation). Comparisons of effects among the pirfenidone-treated groups and the control groups were performed by one-way analysis of variance (ANOVA) with Bonferroni correction. For all statistical analyses, the level of significance was set at P<0.05.

## Results

### Human pterygium fibroblasts (HPFs) culture and identification

**Figure 1:**
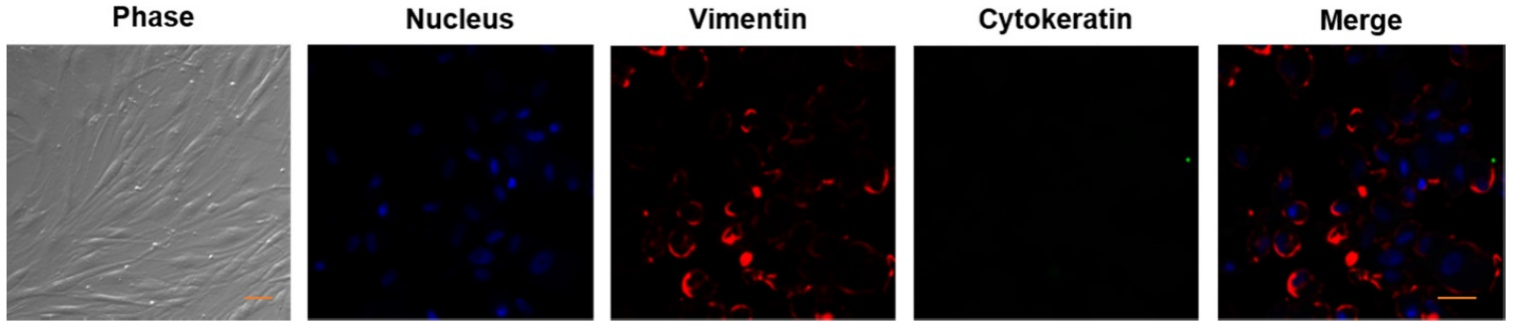
** Verification of HPFs by phase contrast and immunocytochemistry staining for stromal marker vimentin and not for epithelial marker cytokeratin.** Phase contrast showed that the proliferating cells appeared as spindle shaped morphology with numerous cytoplasmic processes forming a network of intercellular contacts. The fibroblast-like stromal morphology of HPFs was further confirmed by immunocytochemistry staining for stromal marker vimentin and not for epithelial marker cytokeratin. Scale-bar: 50 µm.

As mentioned in the Methods, we established the primary cultures of HPFs from pterygia surgically removed from patients. After in vitro culturing for 7 to 14 days, the proliferating cells appeared in spindle shape with a network of intercellular contacts (Figure 1). The fibroblast-like morphology of HPFs was further confirmed by immunocytochemistry staining for stromal marker vimentin and not epithelial cytokeratin (Figure 1).

### Pirfenidone inhibited proliferation of human pterygium fibroblasts without significant cytotoxicity at 0.2 mg/ml

**Figure 2:**
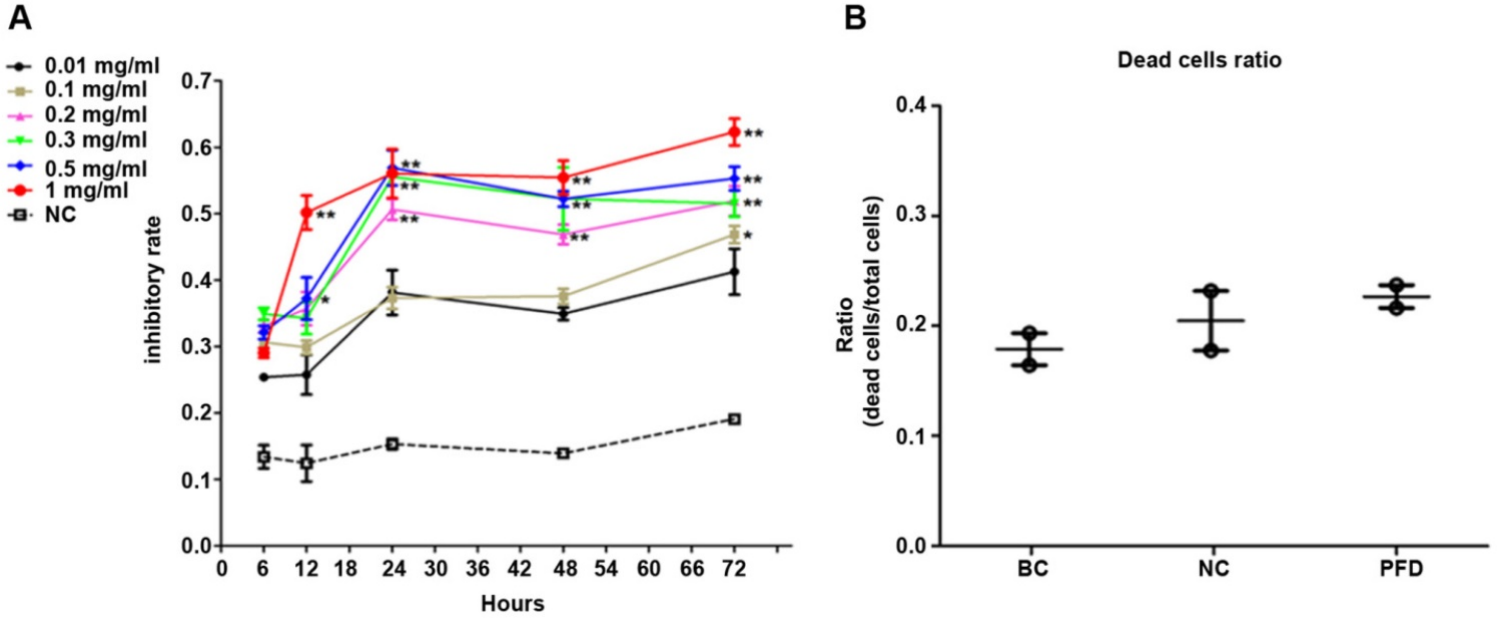
** Pirfenidone inhibited the proliferation of HPFs and had no significant cytotoxicity at 0.2 mg/ml. (A)** The curve for cell growth inhibition rate. HPFs cultured with the complete medium were treated with different concentrations of pirfenidone for 6, 12, 24, 48 and 72 h. The cell growth inhibitory rates were determined by MTT assay, as described in the Material and Methods part. The inhibitory rates showed a dose-dependent increase. Data are representative results from three independent experiments. n=3 for each group. *P<0.05, and **P<0.001, versus (vs.) the negative control (NC) cells cultured in the complete medium without pirfenidone at the indicated time points. **(B)** The trypan blue exclusion test in HPFs treated with 0.2mg/ml pirfenidone for 24 hours. There was no apparent alteration of dead cells ratio between the blank cell (BC, HPFs cultured in complete culture medium) group, the negative control (NC, HPFs treated with carboxymethylcellulose solution) group and the pirfenidone-treated (PFD, HPFs treated with 0.2mg/ml pirfenidone) group. Data are representative results of three independent experiments. n= 6 for each group.

We used MTT assay to detect the effect of pirfenidone on established HPFs proliferation. When compared with the negative control (NC, without pirfenidone in the culture medium) group, all pirfenidone-treated groups had significant inhibition of proliferation at all time points (Figure 2A). Particularly, the proliferation inhibitory rate of HPFs in the 0.2 mg/ml pirfenidone group reached approximately 50% even after treatment for 24 hours, which was significantly different from the control group (P<0.01). The reduction in proliferation was more profound in the groups with 0.3, 0.5 and 1 mg/ml pirfenidone at 24, 48, and 72 hours (P<0.01). The anti-proliferative effect of pirfenidone was prominent starting at the concentration of 0.2 mg/ml at 24 hours and reached the plateau when pirfenidone was at a concentration of 0.3 mg/ml (Figure 2A). Moreover, 24 hours after treatment, 0.2 mg/ml pirfenidone did not display cytotoxicity on HPFs, as evidenced by similar trypan blue exclusion rates among the blank control group (15.04%); negative control group (21.00%) and PFD group (24.95%) (Figure 2B). Thus, the dose of 0.2 mg/ml pirfenidone was chosen for later experiments and the observation period of 24 hours was used in subsequent experiments.

### Pirfenidone induced G0/G1 arrest and inhibited motility of HPFs

**Figure 3:**
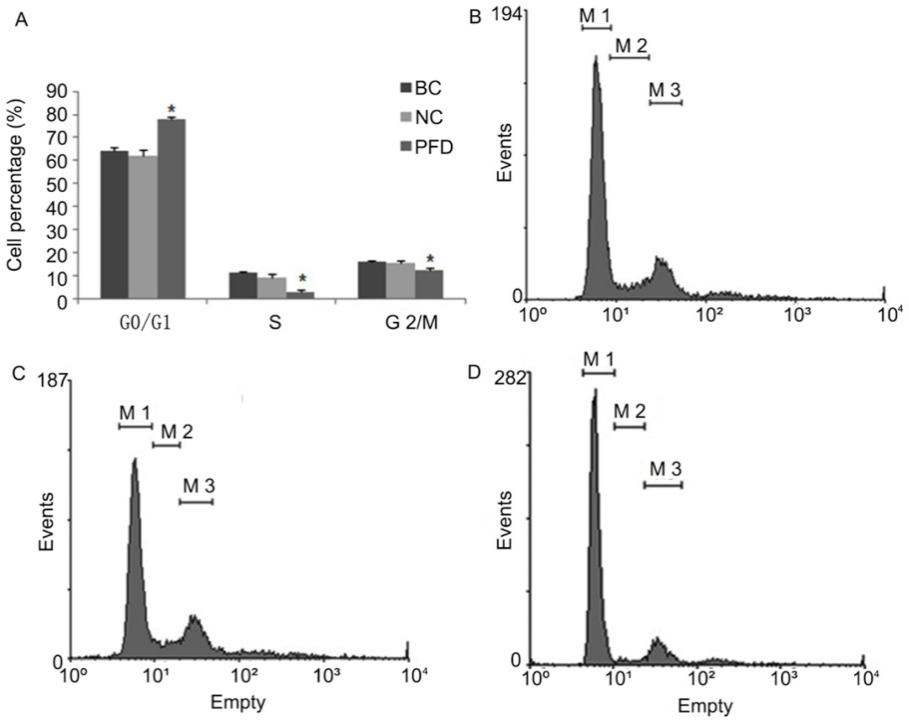
** Pirfenidone induced G0/G1 arrest in HPFs. (A)** Summary of the distribution of cell cycle phases in HPFs treated with or without pirfenidone. n=3 for each group. BC, blank control; NC, negative control; PFD, pirfenidone. **(B-D)** Representative flow cytometry profile showing the distribution of cell cycle phases in indicated groups. Compared with the BC **(B)** and NC **(C)** groups, 0.2mg/ml pirfenidone **(D)** arrested HPFs at the G0/G1 phase. As a result, the percentage of cells in the S and G2/M phase decreased. Data are representative results from three independent experiments. *P <0.05 versus BC and NC groups. M1, G0/G1 phase; M2, S phase; M3, G2/M phase.

We also evaluated the impact of pirfenidone on the distribution of cell cycle phases in HPFs by flow cytometry. As shown in Figure 3, at 24 hours after treatment with 0.2 mg/ml pirfenidone, HPFs had significantly increased proportion of cells at the G0/G1 phase, when compared with the blank control and negative control groups. Correspondingly, the proportion of HPFs in S phase and G2/M phase significantly decreased upon pirfenidone treatment (P<0.05). These results indicated that the anti-proliferative effect of pirfenidone at the concentration of 0.2 mg/ml was associated with G0/G1 cell cycle arrest in HPFs.

### Pirfenidone exposure significantly inhibited migration of HPFs in scratch assays and collagen contraction of HPFs in FPLC assays

**Figure 4:**
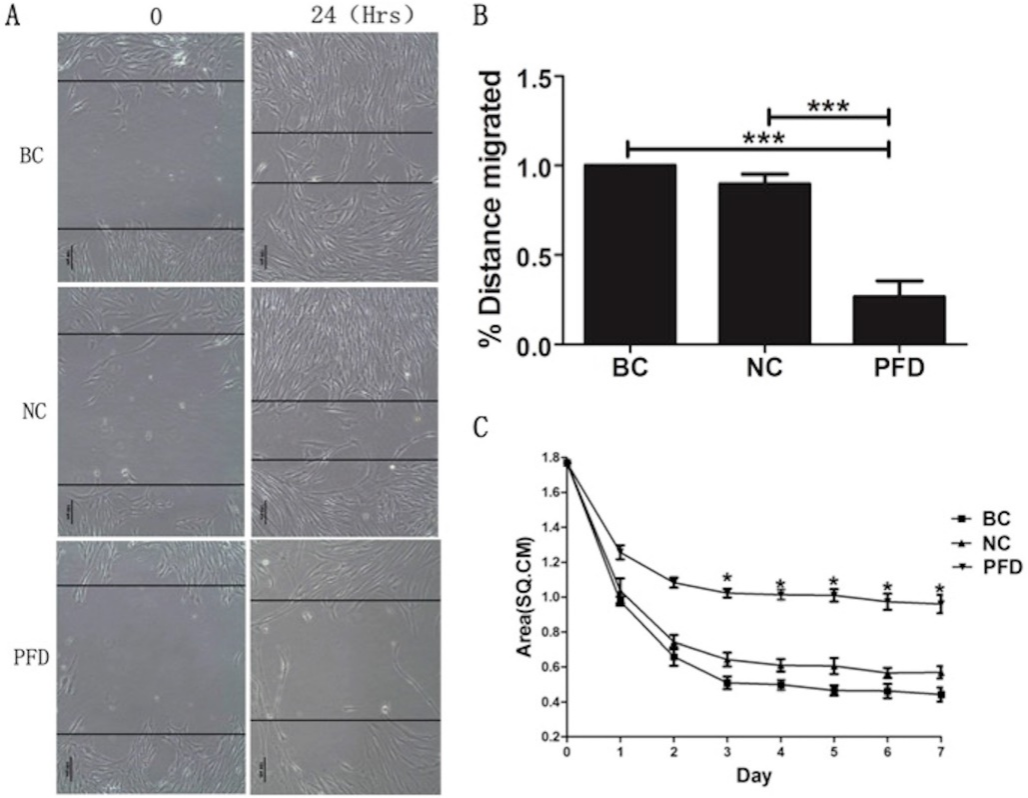
**Pirfenidone inhibited the migration of HPFs in scratch assays and the collagen contraction of HPFs in the FPLC assays. (A)** Representative images showed decreased migratory ability of HPFs at 24 hours after scratches were applied to HPFs that were treated with 0.2mg/ml pirfenidone. Scale bar = 100 µm. **(B)** Summarized migration distances of HPFs in scratch assays. **(C)** HPFs attached to collagen gels were cultured for 24 h under the indicated culture conditions. The data presented represent the range of FPCL responses area for three groups. Data are expressed as the mean ± SD of results obtained from triplicate cultures under each condition. *P<0.05. BC, blank control; NC, negative control; PFD, pirfenidone.

The effect of pirfenidone on the migration of HPFs was evaluated by the scarification test. While HPFs in the blank control (391.731±7.952μm) and negative control group (350.878±6.948μm) demonstrated evident migration for scarification repair, HPFs upon pirfenidone treatment (107.923±10.451μm) showed significantly reduced migration distance (Figure 4A, B). Therefore, pirfenidone at the concentration of 0.2mg/ml can significantly reduce the motility of HPFs.

To investigate the molecular and cellular mechanisms involved in pirfenidone-mediated attenuation of pterygium fibrosis, we examined the effect of pirfenidone on fibroblast-mediated collagen contraction (FPLC) by FPLC assays as an in vitro model of scar collagen remodeling. As shown in Figure 4C, the mean diameter of FPCL area in pirfenidone-stimulated HPFs was 1.77 cm, 1.24 cm, 0.92 cm, 0.82 cm, 0.81 cm, 0.80 cm, 0.74 cm and 0.72 cm at day 0 to day 7, respectively. Compared with the blank control and negative control group, exposure to pirfenidone caused significant inhibition of collagen contraction of HPFs by FPLC assays starting at day 3 after treatment (P<0.05).

### Pirfenidone treatment significantly reduced the expression of TGF-β1, TGF-β2 and MMP1 but not TIMP1 in HPFs, which medicates cell proliferation and migration

**Figure 5:**
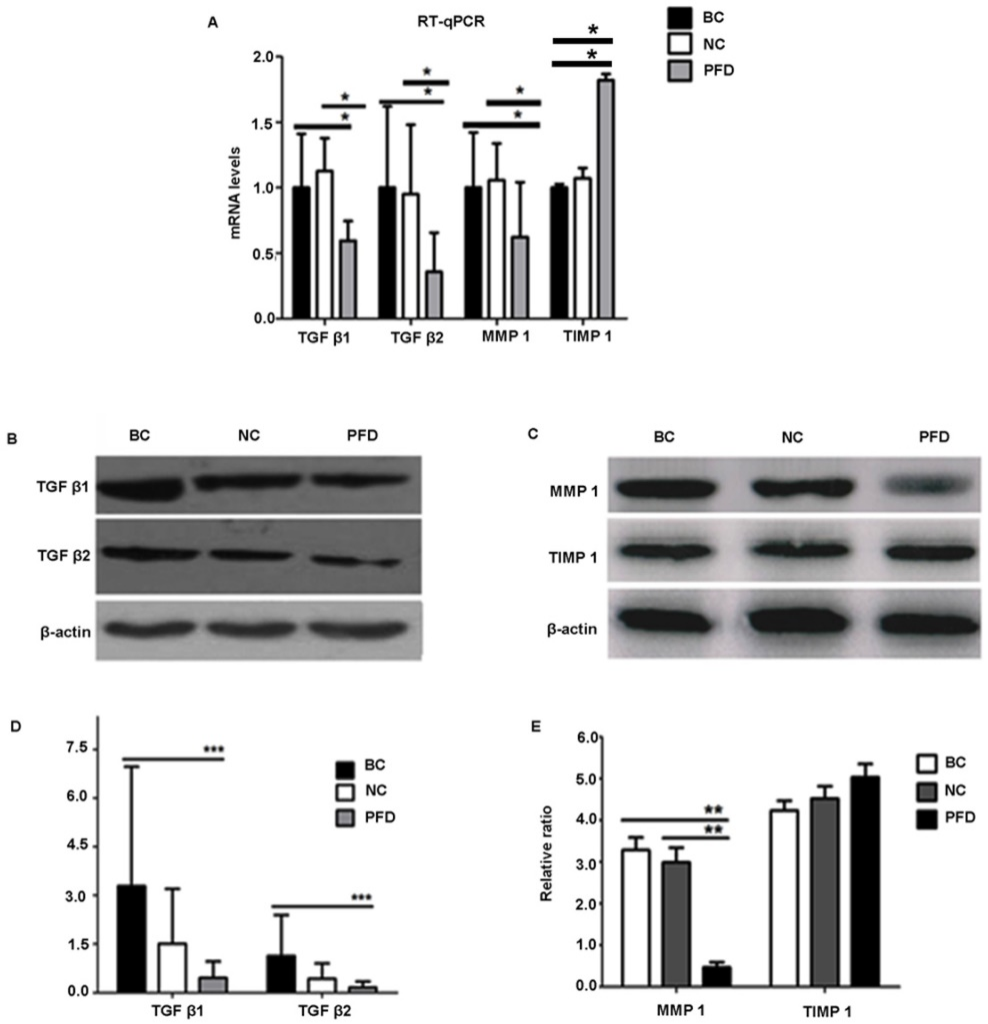
** Pirfenidone treatment significantly reduced the mRNA and protein expression of TGF-β1, TGF-β2 and MMP1 in HPFs, but exerted no effects on TIMP1 expression. (A)** Pirfenidone (0.2mg/ml) decreased mRNA expression of TGF-β1, TGF-β2 and MMP-1, when compared with the BC and NC groups. The TIMP-1 mRNA level was statistically different among the three groups, however, its protein level was not **(C)**. Data are representative results from three independent experiments. n=3 for each group. *P<0.05, vs the BC or NC group as indicated. BC, blank control; NC, negative control; PFD, pirfenidone. **(B-E)** Western blot showed that 0.2mg/ml pirfenidone reduced the protein levels of TGF-β1, TGF-β-2 and MMP-1, when compared to the BC and NC groups. The TIMP-1 protein level was not statistically different among three groups. Representative images with targeted bands (B, C) and summarized data **(D, E)** are shown. Data are representative results from three independent experiments. n=3 for each group. (**: P<0.001, ***: P<0.0001, vs. the BC or NC group as indicated).

To further elucidate the mechanisms underlying the potentially protective effect of pirfenidone in HPFs, we examined the expression levels of fibrosis-associated molecules, including TGF-β1, TGF-β2, MMP-1 and TIMP-1. Our RT-qPCR and western blot results showed similar trends on expression levels of these molecules (Figure 5). Compared with the blank control and negative control groups, HPFs treated with 0.2mg/ml pirfenidone for 24 hours had significantly down-regulated expression of TGF-β1, TGF-β2 and MMP-1. However, there was no statistically significant difference for both the mRNA expression (Figure 5A) and protein expression (Figure 5D, 5E) of TIMP-1 among the three groups.

**Figure 6:**
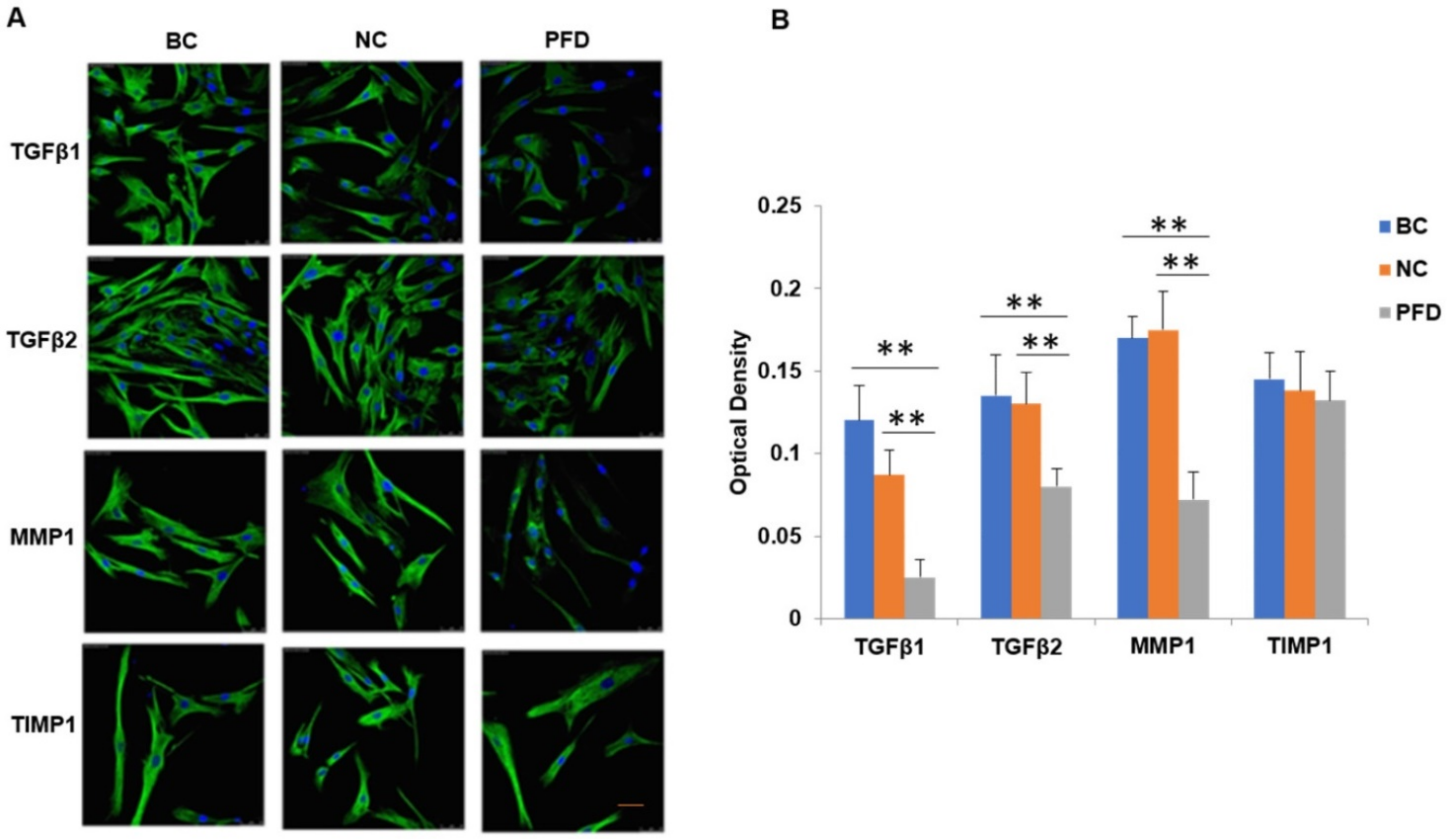
**Immunofluorescent detection of TGF-β1, TGF-β2, MMP1 and TIMP1 in HPFs. (A)** Representative immunofluorescence staining images showed the distribution of TGF-β1, TGF-β2, MMP1 and TIMP1 proteins in the cytoplasm and nuclei of HPFs in the indicated groups. Relatively moderate staining was present for TGF-β1, TGF-β2 and MMP1 in the BC and NC groups, while the treatment with 0.2mg/ml pirfenidone for 24 hours reduced the staining of these molecules. The expression of TIMP1 was not statistically different between three groups (P>0.05). Scale bars, 50 µm. Magnification: ×40. (B) Quantitative results of TGF-β1, TGF-β2, MMP1 and TIMP1 expression in the immunofluorescence staining images. n=6 for each group. **:P<0.001, vs. the BC or NC group as indicated. Bar=50 µm.

**Figure 7:**
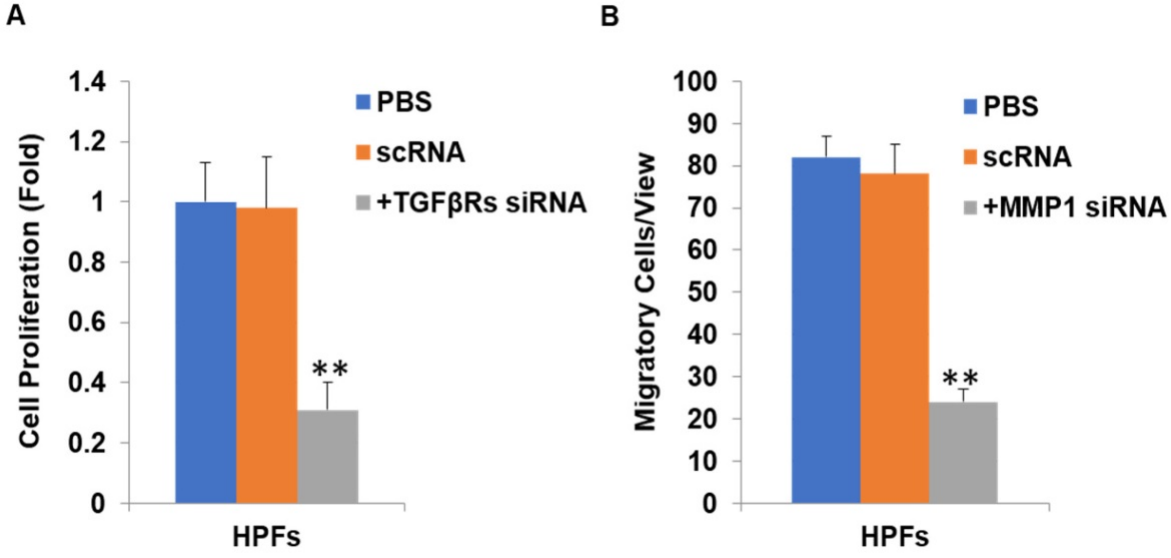
** Inhibition of TGFβ signaling and MMP1 decreases cell proliferation and migration by their siRNA(s) respectively.** After TGFβRs knockdown, the cell proliferation was significantly downregulated (P<0.01, A). In addition, after MMP1 knockdown, the cell migration was significantly downregulated (P<0.01, B).

We further substantiated the expression patterns of TGF-β1, TGF-β2, MMP-1 and TIMP-1 using immunofluorescence staining. Consistent with the results from RT-qPCR and western-blot, TGF-β1, TGF-β2, MMP-1 and TIMP-1 exhibited similar expression patterns in HPFs exposed to 0.2mg/ml pirfenidone for 24 hours (Figure 6). Relatively less staining was identified for TGF-β1, TGF-β2 and MMP1 in pirfenidone-treated HPFs, when compared to the blank control and negative control groups (P<0.05). Similarly, there was no statistically significant difference for the staining intensity of TIMP-1 among the three groups (all P>0.05, Figure 6). Interestingly, inhibition of TGF-β signaling by TGFβRs siRNA inhibited cell proliferation and inhibition of MMP-1 by MMP1 siRNA reduced cell migration significantly (all P<0.01, Figure 7).

## Discussion

The purpose of this study was to find non-toxic antifibrotic agents which can prevent pterygium growth at an early stage and decrease recurrence of pterygium after surgery. The antifibrotic effect of pirfenidone was examined in terms of cell proliferation, cell cycle progress, migration, and collagen contraction of HPFs, a pivotal cell type implicated in the pathogenesis of pterygium. Our results demonstrated a direct suppressive effect of pirfenidone on cultured HPFs (Figure 2-5), which was associated with decreased expression of TGF-β1, TGF-β2 and MMP-1 (Figure 6), similar to what has been previously reported in other tissues such as lung 19, 20, liver 21 and kidney 16. Indeed, pirfenidone may exert its function by inhibiting TGF-β and MMP-1 signaling.

Pirfenidone is an anti-inflammatory and antifibrotic agent with inhibitory effects on several cell types *in vitro*, including hepatic stellate cells, renal and intestinal fibroblasts, myometrial and leiomyoma cells 16,30-32. Although pirfenidone has been widely used, its potential mechanism has not been fully understood. The anti-fibrosis mechanisms of pirfenidone have been linked to signaling regulation of multiple cytokines, such as TGF-β, PDGF, TIMP-1, and MMPs 15,33. For example, Kim* et al.* reported that pirfenidone at 1.9 mg/ml inhibited the growth of orbital fibroblasts obtained from patients with thyroid-associated ophthalmopathy (TAO) 34. Choi *et al.* proved the inhibitory effect of pirfenidone (from 0.25 to 0.5 mg/ml) on TGF-β-mediated fibrogenesis in the human RPE cell line ARPE-19 35. In our previous study, we have shown that pirfenidone had an inhibitory effect on proliferation, migration and collagen contraction of Tenon's fibroblasts *in vitro* and *ex-vivo*. Its anti-fibrosis properties are related to the regulation of TGF-β mRNA and protein expression reported by us 23,24. Signaling from TGF-β1, TGF-β2 and MMP-1 is also correlated with the proliferation and fibrosis of fibroblasts under pathological conditions, such as fibrosis of liver, lung, and kidney 36,37. The effects of pirfenidone in this study were closely associated with down-regulated expression of TGF-β (Figure 6), instead of a toxic effect. Trypan blue exclusion test showed that, compared with the control group, the level of pirfenidone used in this study had no obvious cytotoxic effect (Figure 2). These findings raise the possibility that pirfenidone may represent a new therapeutic agent for pterygium. In our previous study, we also investigated the pharmacokinetics of topically administered pirfenidone (0.5%) in rabbit eyes 24, 38. The mean maximum concentration (C_max_) of pirfenidone in conjunctiva was 9.62 mg/g and the half-life for conjunctiva was 34.16 min 24, 38. Therefore, topical administration of pirfenidone may be an effective and safe approach to inhibit the growth or recurrence of pterygium.

The pathological characteristics of pterygium are limbic cell proliferation, inflammatory infiltrates, fibrosis, angiogenesis and destruction of extracellular matrix 2,39,40. Although some hypotheses are considered to be part of the pathogenesis of pterygium, including genetic susceptibility, anti-apoptosis mechanism, cytokines, growth factors, extracellular matrix remodeling, immune mechanism and viral infection, the formation mechanism of pterygium is still not completely clear 41. Some potential fibrogenic and angiogenic growth factors, such as TGF-β, bFGF (basic fibroblast growth factor) and PDGF, have been found in pterygium tissues by immunohistochemical methods. MMP-1 is one of the most abundant subtypes of MMPs in pterygium 42. It is possible that matrix-bound MMP-1 forms a reservoir of latent enzyme in pterygium epithelial cells. When exposed to ultraviolet light stimulation, MMP-1 increases in dose and time dependence, and releases chemotactic collagen peptide, promoting leukocyte infiltration and inflammation. This response pattern of MMP-1 resembles the disorder model of wound healing 43. TIMP-1 is an inhibitor of MMP-1 and is normally co-expressed with MMP-1 and inhibits the active forms of MMP-1. In pterygium, the overexpression of MMP-1 breaks the balance between MMP-1 and TIMP-1, and the imbalance may cause pterygium to infiltrate through the normal cornea 44,45.

In the current study, we found that pirfenidone suppressed the expression of TGF-β1, TGF-β2 and MMP-1 in mRNA and protein levels (Figure 6). However, there was no significant difference in the transcript and protein expression of TIMP-1 among three groups (Figure 6). Confocal images from the immunofluorescence staining showed that TGF-β1, TGF-β2, MMP-1 and TIMP-1 were all expressed in the cytoplasm and nucleus of HPFs (Figure 6). That is, pirfenidone significantly reduced the expression of TGF-β1, TGF-β2, and MMP-1 in HPFs but did not alter the expression of TIMP-1. These findings suggest that the TGF-βs and MMP-1/TIMP-1 signaling pathways are likely involved in the antifibrotic mechanism of pirfenidone in HPFs. Recently, a similar study from Lee et al. also highlighted that the antifibrotic effect of pirfenidone on HPFs was associated with decreased TGF-β expression 46. In their study, it was also found that pirfenidone had a significant inhibitory effect on HPF proliferation, migration and collagen synthesis. However, unlike our MTT assay results, they did not observe differences between cells treated with other concentrations of pirfenidone (0.5, 1.0, and 1.5 mg/ml) versus control cells 46, which might be due to different sources and purity of pirfenidone, as well as other varied experimental conditions like the in vitro model of HPFs culture. Strikingly, inhibition of TGF-β signaling by TGFβRs siRNA inhibited cell proliferation and inhibition of MMP-1 by MMP-1 siRNA reduced cell migration significantly (all P<0.01, Figure 7), suggesting that inhibition of cell proliferation is medicated by inhibition of TGF-β signaling while reduction of cell migration is regulated by inhibition of MMP-1. Our studies also suggest that pirfenidone is a safe adjuvant for pterygium surgery to prevent its recurrence.

In conclusion, our results showed that pirfenidone has significant inhibitory effects on proliferation, migration, and collagen contraction of primary cultured HPFs, and the anti-fibrotic properties of pirfenidone are related to the downregulation of TGF-β1, TGF-β2 and MMP-1. Thus, we conclude that pirfenidone is a promising antifibrotic agent for inhibiting pterygium growth and preventing pterygium recurrence after excision.

## Figures and Tables

**Table 1:** The sequences of DNA primers used in this study

Gene	Primer sequence (5'-3')	Products size (bp)
human TGFβ-1	F: CAGCAACAATTCCTGGCGATAC	139bp
	R: GCTAAGGCGAAAGCCCTCAAT	
human TGFβ-2	F: GTCATACCACCTTTCCGATTGC	125bp
	R: GACGGCACAGGGATTTCTTCTA	
human MMP-1	F: TGAAGAATGATGGGAGGCAAGT	123bp
	R: TCAGGGTTTCAGCATCTGGTTT	
human TIMP-1	F: CCTGTTGTTGCTGTGGCTGAT	272bp
	R: GGTTGTGGGACCTGTGGAAGTA	
human β-actin	F: AGTTGCGTTACACCCTTTCTTG	150bp
	R: TCACCTTCACCGTTCCAGTTT	

## Abbreviations

PFD: pirfenidone
HPF: human pterygium fibroblast
IC50: 50% inhibiting concentration
MMC: mitomycin C
TGF-β: transforming growth factor beta
CTGF: connective tissue growth factor
PDGF: platelet-derived growth factor
TNF-α): TNF-α, tumor necrosis factor
RPE: retinal pigment epithelial
KMU: Kunming Medical University
HE: hematoxylin-eosin
DMSO: dimethyl-sulfoxide
OD: optical density
PI: propidium iodide
FPCL: fibroblast-populated collage lattice
FPLC: fibroblast-mediated collagen contraction
MMP: matrix metalloproteinase
TIMP: tissue inhibitors of metalloproteinases
TAO: thyroid-associated ophthalmopathy
